# High-temperature Mechanical Properties and Their Influence Mechanisms of ZrC-Modified C-SiC Ceramic Matrix Composites up to 1600 °C

**DOI:** 10.3390/ma13071581

**Published:** 2020-03-30

**Authors:** Jianjun Sha, Shouhao Wang, Jixiang Dai, Yufei Zu, Wenqiang Li, Ruyi Sha

**Affiliations:** 1 State Key Laboratory of Structural Analyses for Industrial Equipment, Dalian University of Technology, Dalian 116024, China; jxdai@dlut.edu.cn (J.D.); yfzu@dlut.edu.cn (Y.Z.); leawq@mail.dlut.edu.cn (W.L.); sharuyi@mail.dlut.edu.cn (R.S.); 2 Key Laboratory of Advanced Technology for Aerospace Vehicles, Liaoning Province, Dalian University of Technology, Dalian 116024, China; 3 School of Materials Science and Engineering, Dalian University of Technology, Dalian 116024, China; wangshouhao@mail.dlut.edu.cn

**Keywords:** ceramic matrix composite, high-temperature mechanical properties, microstructure evolution, UHTC nanoparticle, thermally-induced residual stress

## Abstract

In order to understand the influence of the mechanisms of ZrC nanoparticles on the high-temperature mechanical properties of C-SiC ceramic matrix composites, the mechanical properties were measured from room temperature (RT) to 1600 °C under vacuum. The microstructures features were characterized by scanning electron microscopy. In comparison with the composites without ZrC nanoparticles, the ZrC-modified composite presented better mechanical properties at all temperatures, indicating that the mechanical properties could be improved by the ZrC nanoparticles. The ZrC nanoparticles could reduce the residual silicon and improve the microstructure integrity of composite. Furthermore, the variation of flexural strength and the flexural modulus showed an asynchronous trend with the increase of temperature. The flexural strength reached the maximum value at 1200 °C, but the highest elastic modulus was obtained at 800 °C. The strength increase was ascribed to the decrease of the thermally-induced residual stresses. The degradation of mechanical properties was observed at 1600 °C because of the microstructure deterioration and the formation of strongly bonded fiber–matrix interface. Therefore, it is concluded that the high temperature mechanical properties under vacuum were related to the consisting phase, the matrix microstructure, and the thermally-induced residual stresses.

## 1. Introduction

Carbon fiber-reinforced silicon carbide-based matrix (C/C-SiC) composites have been attracting increasing attention in the development of aerospace technology because of their lightweight, superior high-temperature mechanical properties, good oxidation resistance, and excellent thermal shock resistance [1,2,3,4,5,6]. However, with the persistent pursuit of improving the performance of hot structures, further requirements have been put forward to enhance the comprehensive performance of the carbon fiber-reinforced ceramic matrix composites, which helps to fulfill the engineering application under harsh environments.

The introduction of the ultra-high temperature ceramics (UHTCs) into matrix is a favorite approach [7,8,9,10]. Among the different UHTCs, ZrC and SiC are superior candidates due to their good endurability in high temperature environment, such as good anti-oxidation resistance and anti-ablation properties [11,12]. In particular, much attention was paid to ZrC ceramic due to its lightweight, good chemical inertness, high melting temperature (3540 °C) and even for its oxide (ZrO_2_: 2770 °C). Furthermore, the coexistence of ZrC and SiC in carbon fiber-reinforced ceramic composites can significantly improve the oxidation resistance because of the generation of the in-situ molten binary oxides in oxidative environments, which can act as the self-healing sealant to seal the cracks/pores, and protect the internal matrix and reinforcing fibers.

Recently, some investigations were carried out to introduce UHTCs into ceramic-based matrix composites via various techniques [13,14,15,16]. Because fine ceramic particles with a relatively high volume fraction can easily infiltrate into the intra-fiber bundles and produce a homogeneous distribution in composites, the slurry infiltration (SI) could be an available approach for introducing the UHTCs into the ceramic-based matrix composites [15,16]. 

By far, most researches have focused on the room-temperature mechanical performance, oxidation behavior, ablative resistance as well as the related mechanisms [17,18]. Actually, the process-related microstructure, the matrix phase and the service environments can significantly affect the high-temperature mechanical properties. In particular, under the high temperature oxidative environments, it is difficult to understand the intrinsic influence mechanisms (related to the microstructure evolution and the internal stress variation etc.) on the mechanical properties of composites because of the multi-mechanisms activated in such environment. Therefore, the mechanical behaviors and influence mechanisms of the composites exposed to high temperature in the absence of oxidation are indispensable, which can effectively reveal the influence of the microstructure evolution and the internal stress variation on mechanical properties of composites to some extent. 

In previous work [19], it was found the introduction of ZrC could improve the anti-oxidation resistance and the high temperature mechanical properties of carbon fiber-reinforced C-SiC matrix composites under oxidative environment. However, due to the obvious oxidation at high temperature, it is difficult to understand how the intrinsic microstructure, matrix phase and the internal stress variation impact the mechanical properties. Therefore, in the current work, in order to know the high temperature mechanical properties and their influence mechanisms of ZrC nanoparticle-modified C-SiC ceramic matrix composite, where the composites were fabricated by a hybrid process of slurry infiltration (SI) and liquid silicon infiltration (LSI). The mechanical properties as a function of temperature were evaluated from RT to 1600 °C under vacuum. Field-emission scanning electron microscopy (FE-SEM) was employed to characterize the microstructure features of composites before and after high-temperature mechanical tests. Moreover, combining the mechanical properties with the microstructures and the analysis of residual stresses, the intrinsic mechanisms on the mechanical properties were analyzed and discussed.

## 2. Experimental

### 2.1. Materials

In order to fabricate the ZrC modified C-SiC-based composite, 2D carbon fabrics (T700, Toray, Tokyo, Japan) were used as the reinforcements. The density of the fabrics is about 1.80 g/cm^3^. ZrC nanoparticles (average size: 60 nm, density: 6.73 g/cm^3^, purity: 99.8%, Hefei Kaier nanometer technology, China) were utilized to modify the microstructure and mechanical properties. Phenolic resin was used as the precursor for the production of pyrolytic carbon.

### 2.2. Preparation of ZrC Slurry

The preparation of ZrC slurry was as follows. Firstly, the ZrC nanoparticles were dispersed in a colloidal solution. And then the saturated phenolic solution was prepared by dissolving the phenolic resin into the alcohol. The ZrC slurry was prepared by adding the ZrC-containing colloidal solution into the phenolic solution in batches while stirring. The mass ratio of ZrC to phenolic resin was adjusted to 1:10.

### 2.3. Preparation of ZrC-modified C-SiC Composites

In order to obtain the green bodies for the fabrication of ZrC-modified C-SiC composites, the carbon fabrics in a vessel were firstly vacuum impregnated by the above-mentioned ZrC slurry. After the impregnation, the fabrics with ZrC slurry were cured and post-cured at 240 °C for 24 h. To convert resin into carbon matrix, the green bodies were carbonized at temperature above 1200 °C in nitrogen atmosphere (purity: 99.9%). The final char yield from the pyrolysis of phenolic resin was about 65% measured by TG test [20]. In this way, the porous ZrC-containing C/C preforms were prepared. For the LSI process, the detailed technical route can be found in the previous work [19]. Firstly, the porous ZrC-modified C/C preforms and a certain amount of silicon powders were put into a graphite crucible. And then the graphite crucible was carefully moved into the chamber of furnace. Secondly, the furnace was heated to above 1500 °C under vacuum and the silicon melt was formed. The silicon melt with a low viscosity climbed into the pores of ZrC-modified C/C preforms by capillary force, and simultaneously the SiC-based matrix was formed by in-situ reaction of liquid Si with adjacent carbonaceous materials. A similar process for the formation of SiC can also be found in the literature [21,22,23]. Following this procedure, the ZrC-modified C-SiC composites were fabricated. Furthermore, the C-SiC composites without ZrC nanoparticles were also fabricated by the same processes as a counterpart for comparison. For convenience, hereafter, the C-SiC composites without and with ZrC nanoparticles are referred to as CSZ0 and CSZ10, respectively.

### 2.4. Characterization

The consisting phase of the composites was analyzed by X-ray diffractometer (XRD, Model D/Max 2400, Tokyo, Japan), using Cu Kα radiation (λ = 0.154 nm) at scanning angles ranging from 20 to 80°. Field-emission scanning electron microscopy (FE-SEM, Nano 450, Hillsboro, OR, USA) was used to characterize the microstructure of finely polished surface and the fracture surface of the composites before and after the mechanical tests. To investigate the influence of ZrC on the high-temperature mechanical properties of C/C-SiC composites, the mechanical properties on a rectangular bar were measured by using three-point flexural test according to ASTM C-1341 at temperatures ranging from RT to 1600 °C under vacuum (about 1 Pa) on a universal testing machine (Changchun Fangrui Technology Co., Ltd., Changchun, China). The dimensions of the final specimens were 25 × 4 × 3 mm^3^. The crosshead speed and support span were adjusted to 0.5 mm/min and 20 mm, respectively. The displacement during flexural test was monitored by an extensometer. The inter-laminar shear strength (*ILSS*) was measured by compression of double-notched shear (DNS) specimen at RT. The heating rate was set to be about 15 °C/min. The test was started after the temperature raised to the terminal temperature for 15 min under vacuum. At least three samples were measured to calculate the mean value for each condition. The high-temperature flexural strength (*S_f_*), elastic modulus (*E_f_*) and *ILSS* of composites were calculated by the following Equations (1)–(3):

(1) $$S_{f}=\frac{3PL}{2bd^{2}}$$

(2) $$E_{f}=\frac{kL^{3}}{4bd^{3}}$$

(3) $$ILSS=\frac{P}{bh}$$

where *P* is the maximum load, *L* the span of the composite, *b* the width of the specimen, *d* the height of the specimen, *k* the slope of load-displacement curve, and *h* the distance between notches.

## 3. Results and Discussion

### 3.1. Influence of ZrC Nanoparticles on the Morphologies of C/C Preforms

**Figure 1:**
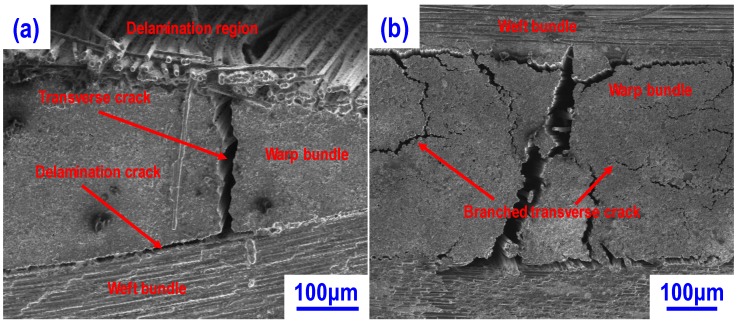
Surface morphologies of C/C preforms: (**a**) C/C preform without ZrC nanoparticles; (**b**) C/C preform with ZrC nanoparticles.

Figure 1 shows the morphologies of C/C preforms without and with ZrC nanoparticles. It can be seen that their morphologies are clearly different. In the C/C preform without ZrC (Figure 1a), the transverse and delamination cracks presented as the main microstructure feature. The transverse cracks located in the warp fiber bundle region and their propagation path inclined to be perpendicular to the weft fiber bundles. The delamination cracks presented at the interface between the weft and warp fiber bundles, being connected with the transverse cracks, resulting in the formation of several C/C segments. As for the C/C preform with ZrC nanoparticles (Figure 1b), the branched transverse cracks, which located in the warp fiber bundle region, were the main microstructure feature. The turning of the crack propagation path in the warp fiber bundle region could be related to the change of interfacial bonding strength between fiber and resin matrix in the green bodies. In our previous works [24,25], it has evidenced that such interfacial bonding strength can significantly affect the morphology of C/C preforms. For the green body with low interfacial bonding strength, during the carbonization stage, it was easy to turn the crack propagation path. This is quite possible in the case of present work due to the deposition of ZrC nanoparticles on the surface of carbon fibers. The deposition of ZrC nanoparticles on the carbon fibers’ surface could reduce the effective contact area between carbon fibers and resin matrix, leading to a low interfacial bonding strength. During the carbonization process, the green body with low fiber-resin bonding strength would result in the branched transverse cracks in the C/C preform (Figure 1b). The measured open porosities were 30.2% and 39.3% for the C/C preforms without and with ZrC nanoparticles, respectively. The open pores that acted as the channels for the silicon melt infiltration can affect the microstructure, matrix composition, and mechanical properties of C/C-SiC composites.

**Figure 2:**
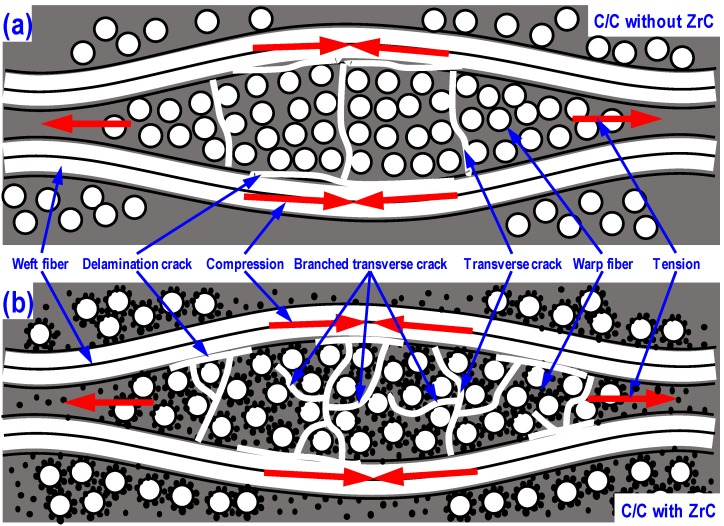
Formation mechanism for the porous structure of C/C: (**a**) C/C preform without ZrC nanoparticles; (**b**) C/C preform with ZrC nanoparticles.

Figure 2 shows the formation mechanisms for the different morphologies of C/C preforms. During the conversion of green bodies to the C/C preforms, the resin matrix in the warp fiber bundle region was subjected to a significant volume shrinkage, while the fiber in the weft direction (very low coefficient of thermal expansion for carbon fiber along the axial direction) impeded the shrinkage of the resin matrix within the warp fiber region. Such interaction generated the tension force in the matrix of the warp fiber bundle region. In contrast, the fibers in the weft direction experienced the compression force. For the green body without ZrC, due to the strong interfacial bonding strength, the shear interaction between weft and warp fiber bundles and the tension force in the warp fiber bundle region would generate the delamination and transverse cracks. Further, the transverse cracks tend to propagate, perpendicularly to the weft fibers under the induction of tension force (Figure 2a). For the C/C preform with ZrC, the interaction between weft and warp fiber bundles can be alleviated by the release of tension force in the warp fiber bundles due to the generation of micro debonding cracks caused by the weak interfacial bonding strength. Moreover, due to the impedance of ZrC nanoparticles and interface debonding, the propagation of the micro cracks in the warp fiber bundle region could easily turn their path to form the branched transverse cracks as shown in Figure 2b. 

### 3.2. Influence of ZrC Nanoparticles on the Consisting Phase and Microstructure

**Figure 3:**
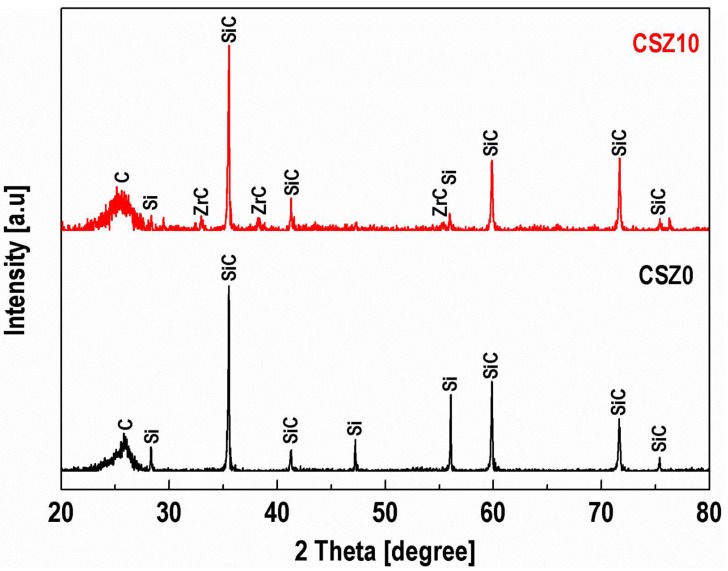
X-ray diffraction spectrum of CSZ0 and CSZ10.

During the LSI process, the silicon carbide matrix was formed in-situ as a result of reaction between molten silicon and carbon. Figure 3 depicts the XRD patterns of the composites CSZ0 and CSZ10. As it can be seen, composite CSZ0 contains carbon and SiC phases, which are corresponding to the carbon fiber and the in-situ formed SiC phase, respectively. Meanwhile, Si diffraction peak is also observed in CSZ0, indicating that some residual Si exists in CSZ0. Comparing with CSZ0, the diffraction peak of ZrC was detected in CSZ10 besides those of carbon and SiC. On the other hand, the intensity of diffraction peak for Si was very weak in CSZ10, which means that the introduction of ZrC nanoparticles can reduce the content residual silicon. The occurrence of residual silicon definitely associated with size of infiltration channels (cracks/pores) in C/C preforms. The existence of the residual silicon would limit the use in high-temperature applications due to the poor mechanical properties of silicon [26]. 

**Figure 4:**
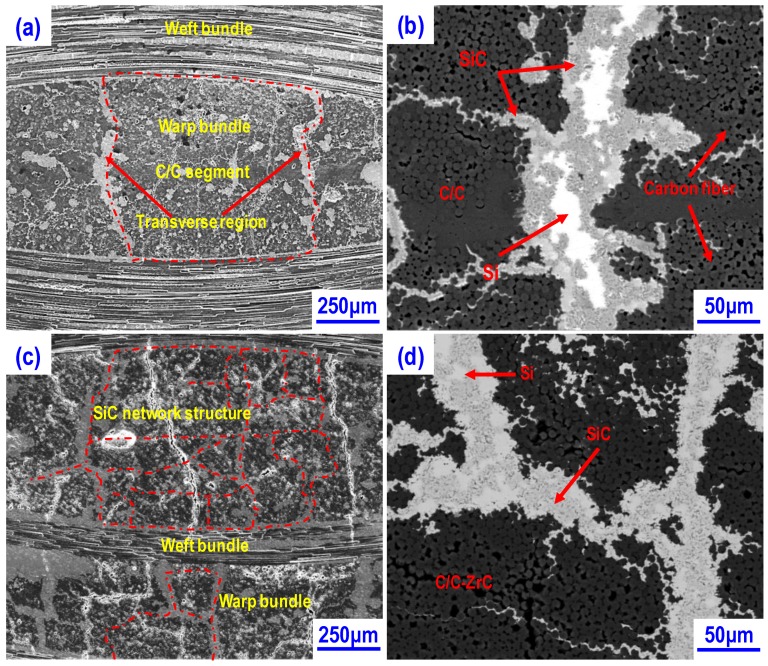
Polished surface morphologies of final ceramic composites: (**a**) SEM cross-section image for CSZ0; (**b**) BSE image for CSZ0 from the selected area in (**a**); (**c**) SEM cross-section image for CSZ10; (**d**) BSE image for CSZ10 from the selected area in (**c**).

The SiC matrix formed by in-situ reaction was further confirmed in both composites by SEM analysis. Figure 4 shows the typical surface morphologies of CSZ0 and CSZ10, respectively. For CSZ0, in addition to carbon fibers, there are two phases is observed with different contrast in the matrix. As shown in Figure 4a, the dark gray, gray, and light gray regions belong to carbon, SiC, and Si phases, respectively. Particularly, they can be clearly observed from the backscattered electron (BSE) image as shown in Figure 4b. The SiC phase (gray) is around the residual Si phase (light gray). In contrast, for CSZ10, the main consisting phases are C and SiC as shown in Figure 4c,d, which is in agreement with the result of XRD analysis. 

Furthermore, it can be seen that the SiC distributions in CSZ0 and CSZ10 are obviously different. For CSZ0, the SiC/Si matrix mainly distributed in the warp/weft fiber bundle boundaries and the transverse regions within warp fiber bundles (Figure 4a,b). Particularly, the SiC matrix in the transverse regions resulted in the formation of several C/C segments in the warp fiber bundles as shown in Figure 4a. In other words, the C/C segments were wrapped by a massive SiC/Si matrix. Inside of each C/C segment, the arrangement of carbon fibers seems to be very compact, as shown in Figure 4b. Meanwhile, some micro pores can also be observed in CSZ0, and occasionally a small amount of SiC matrix occurred around the fiber in the the C/C segment (Figure 4b).

Compared with CSZ0, the distribution of SiC matrix in CSZ10 formed a continuous network structure within the warp fiber bundle regions as shown in Figure 4c,d. Such a network structure would be helpful to the structure integrity of ceramic matrix composites. It is clear that the introduction of ZrC nanoparticles could modify the microstructure of composite. Particularly, the content of SiC matrix was increased and the distribution homogeneity of SiC was improved. The influence of ZrC nanoparticles on the microstructure of C/C-SiC composites should be associated with the fiber-resin matrix interfacial bonding strength in CFRP green bodies. Due to the SiC matrix was mainly formed by in-situ reaction between molten silicon and carbonaceous materials, the distribution of SiC phase in the composites should be highly related to the microstructure of C/C preforms (see Figure 1). A similar phenomenon was also observed in the literature [24].

In addition, more micro pores were observed in CSZ0 (Figure 4a), indicating that CSZ0 has a higher open porosity than that of CSZ10 (Figure 4c). The open porosity and density of CSZ0 and CSZ10 were measured by means of Archimedes’ method. The open porosities are 2.3% and 1.6% for CSZ0 and CSZ10, respectively. The densities are 1.99 g/cm^3^ and 2.06 g/cm^3^, respectively. Furthermore, based on the chemical analysis, for the composite without ZrC, the volume fractions of SiC, Si and C are 24.4%, 10.7%, and 64.9%, respectively; for composites with ZrC, the volume fractions of ZrC, SiC, Si and C are 1.0%, 35.9%, 4.2%, and 58.9%, respectively. Clearly, the residual silicon is small in the ZrC modified composite.

### 3.3. Influence of ZrC Nanoparticles on High-Temperature Mechanical Properties under Vacuum

**Figure 5:**
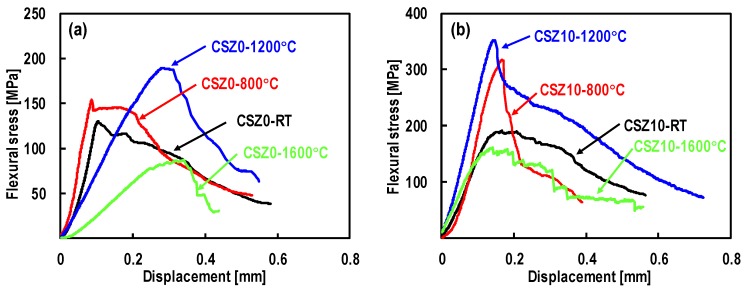
Typical stress-displacement curves of composites under vacuum at temperatures ranging from RT to 1600 °C: (**a**) CSZ0; (**b**) CSZ10.

The mechanical tests were carried out from RT to 1600 °C under vacuum by means of three-point flexural test. Figure 5 shows the typical stress-displacement curves at elevated temperatures.

Based on the observation of stress-displacement curves, both composites exhibited pseudo-plastic deformation behaviors due to the fiber-toughening mechanisms such as interface deboning, fiber sliding and fiber bridging. Also, a liner segment can be observed in the curves before the stress reaching the maximum. After the maximum stress, a stress reduction appeared in the stress-displacement curve corresponding to the occurrence of the first failure of carbon fibers. The survived carbon fibers still enable the composites to tolerate more deformation. This behavior makes the composites superior to monolithic ceramic materials. As it was evident, composite CSZ10 had better mechanical properties, indicating that the mechanical properties were improved by the addition of ZrC nanoparticles. Meanwhile, compared with the deformation behavior at RT, the composites exhibited a better mechanical behavior at 800 °C and 1200 °C. This implied that the toughening effect of fibers at these temperatures became gradually stronger than that at RT.

**Figure 6:**
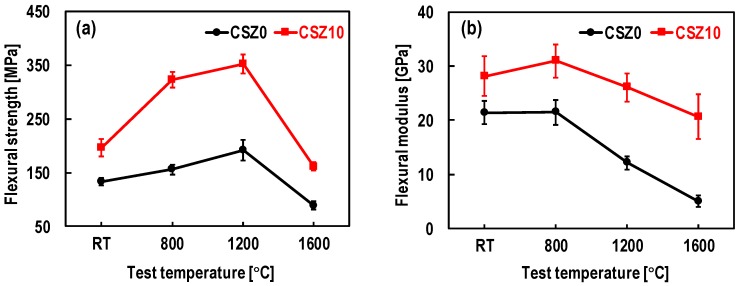
Mechanical properties as a function of temperature under vacuum: (**a**) the flexural strength; (**b**) the flexural modulus.

To clearly observe the variation tendency of mechanical properties of composites, Figure 6 shows the mechanical properties as a function of temperature. The flexural strength of CSZ10 at RT was 196.1 MPa, which was about 47% higher than that of CSZ0 at RT. With the increase of the test temperature, the flexural strengths increased for both CSZ0 and CSZ10 until 1200 °C and then decreased at 1600 °C (Figure 6a). After 1200 °C, the decreased flexural strength might be related to the poor high temperature mechanical property of silicon [26]. The flexural strength at 1600 °C was the lowest, which were 89.0 MPa for CSZ0 and 161.6 MPa for CSZ10, respectively. As for the elastic modulus, they were 21.4 GPa for CSZ0 and 28.1 GPa for CSZ10 at RT, respectively, as shown in Figure 6b. Compared with CSZ0, the introduction of ZrC nanoparticles led to the elastic modulus increased by 31% at RT. At 800 °C, the *E_f_* was the highest, after that it decreased with the increase of the temperature.

Clearly, the change of flexural strength and the flexural modulus presented an asynchronous trend with the temperature increase. At 1200 °C, the flexural modulus for both CSZ0 and CSZ10 decreased, but the flexural strength reached the maximum (see Figure 6). This is because the *E_f_* is more sensitive than the flexural strength to the defects caused by the loading. Similar phenomena were also observed in other work [27].

The strength increase of each composite at temperatures ranging from RT to 1200 °C should be associated with the reduction of the thermally-induced residual stresses accumulated during cooling down from the fabrication temperature to RT. This will be discussed in detail in the following section. From 1200 °C to 1600 °C, the decrease of the elastic modulus for CSZ10 was somewhat slower than that of CSZ0. This indicated that CSZ10 was less sensitive to high temperature than that of CSZ0. The better mechanical properties of CSZ10 than that of CSZ0 may be ascribed to the less residual silicon, homogenous distribution and the network structure of SiC phase, which improved the microstructure integrity of composites (see Figure 4). Furthermore, the homogeneous distribution of SiC phase can also alleviate the stress concentration to some extent. Weak temperature-dependent mechanical properties for CSZ10 means that the introduction of ZrC nanoparticles is favorable to improve the mechanical properties of C/C-SiC composites.

The high-temperature mechanical properties could be related to the consisting phase, the microstructure and the thermal compatibility. Thermally-induced residual stresses caused by poor thermal compatibility [26], could change the internal stress state and the interface bonding of composites.

As we know, all the main consisting phases, including carbon fiber, SiC and ZrC, can endure at very high temperature under nonoxidative environments, which means that the change of high-temperature mechanical properties in the present test conditions is not caused by the intrinsic nature of these consisting phases.

However, the mechanical properties for both CSZ0 and CSZ10 decreased obviously at 1600 °C, which could be mainly ascribed to the residual Si. Since at 1600 °C, the residual silicon became melt (melting point of Si: 1410 °C), so the silicon-filled pockets acted as the defects, which will increase the number and size of critical flaws. During flexural test at 1600 °C, the stress concentration around the defects would occur, and also the strength of fibers would be attenuated due to their reaction with the residual silicon melt. Both would cause the degradation of mechanical properties of composites. However, due to the less residual Si in CSZ10, the retention of mechanical properties was better than that of CSZ0. Therefore, it can be judged that the ZrC nanoparticles can modify the matrix phase and improve the microstructure integrity of the composites.

Secondly, the thermally-induced residual stresses should have an influence on the stress state of fibers and the matrix and the interface bonding, depending on the state and the magnitude of residual stresses [28]. During the cooling down from the fabrication temperature to RT, the thermally-induced residual stresses in the composites were accumulated, resulting in different stress state in different fiber direction because of the mismatch in coefficient of thermal expansion (CTE).

The thermally-induced residual stress plays an important role in the damage (in particular, matrix cracking and fiber-matrix debonding) and the mechanical properties of composites. On a micro-scale level, the thermally-induced stress in composite can be calculated according to the following equation [29]:

(4) $$\sigma_{m}=\frac{V_{f}E_{f}}{1+\frac{E_{f}}{E_{m}}}\left(\alpha_{m}-\alpha_{f}\right)\left(T-T_{f}\right)$$

where *f* and *m* represent the fiber and the matrix, respectively. *E* is the elastic modulus; *V_f_* is the fiber volume fraction in the composite; *α* is the coefficient of thermal expansion; *T* is the test temperature, and *T_f_* is fabrication temperature which can be designed as 1410 °C corresponding to the melting point of silicon.

It is apparent that the thermally-induced residual stress is proportional to Δα·ΔT, where Δ*α* = *α_m_*-*α_f_*, ΔT = *T*−*T_f_*. The CTEs of Si [30], SiC [31], C fiber [19] and ZrC [32] are 4.1 × 10^−6^ K^−1^, 4.6 × 10^−6^ K^−1^, 13 × 10^−6^ K^−1^ in radial direction and 1 × 10^−6^ K^−1^ in axial direction, and 6.7 × 10^−6^ K^−1^, respectively.

In the radial direction, due to the larger CTE of carbon fiber than that of matrix, during the cooling down, the fibers tend to shrink away from the matrix, and the matrix is subjected to tensile stress, which inclines to induce the matrix cracking and interface debonding. Particularly, the mechanical properties of ceramic-based composites are very sensitive to the critical flaws in matrix, which limits the load transferring ability of matrix. When the test temperature is below the infiltration temperature, such tension stress would decrease with the increase of test temperature gradually. Due to the largest ΔT at RT, the thermally-induced residual stress should be the highest. This is the reason why the flaws like micro cracks could be observed in the warp fiber bundle region of as-fabricated composite as shown in Figure 4. However, the matrix is in a compressive state when the temperature is above infiltration temperature.

Assuming that the matrix and the fiber are in good interfacial bonding, considering the thermally-induced residual stress *σ_m_*, when an external tensile stress, *σ_L_*, is applied to the composites, the total stress carried by matrix, *σ_MT_*, can be given theoretically by the following Equation [29]:

(5) $$\sigma_{MT}=\sigma_{m+}\sigma_{ML}=\frac{V_{f}E_{f}}{1+\frac{E_{f}}{E_{m}}}\left(\alpha_{m}-\alpha_{f}\right)\left(T-T_{f}\right)+\frac{E_{m}}{E_{f}+E_{m}}\sigma_{L}$$

where *σ_ML_* is the stress applied to the matrix which is derived from the external applied stress *σ_L_* in composites. Due to the thermally-induced tensile stress in matrix below the fabrication temperature, it is clear, according to Equation (5), that a larger total stress than applied stress in matrix is generated. In contrast, a smaller total stress than applied stress is endured by the matrix when the test temperature is above the fabrication temperature.

According to Equation (4), below the fabrication temperature, the thermally-induced tensile stress *σ_m_* in the matrix decreases gradually with increasing the test temperature due to the decreased ΔT. Using the Equation (4) and parameters of carbon fibers and SiC matrix, the estimated residual stresses *σ_m_* in SiC matrix along the radial direction are 708 MPa at RT, 312 MPa at 800 °C, 107 MPa at 1200 °C and −98 MPa at 1600 °C, respectively. Such residual stresses must be overestimated because they were calculated under an assumption of the perfect interface bonding between fiber and matrix. As we know, in the as-fabricated C/C-SiC composite, micro-cracks can be found everywhere, which can release the thermally-induced residual stress to some extent. Thus, in the real situation, the residual stresses should be lower than above-calculated values. Although the residual stress became small due to the generation of micro-cracks, they were still existing and had an influence on the mechanical properties. Therefore, it is easy to understand that the gradually decreased residual tensile stress *σ_m_* in composites is favorable for increasing the strength when test temperature gradually increased from RT to 1200 °C. Based on the relationship of *σ_MT_* and *σ_ML_* in Equation (5), it is obvious that a smaller external stress is needed to fracture the composite due to the existence of residual tensile stress. Therefore, the composite would be failed under a smaller external stress in case of the larger thermal tensile residual stress existed in the matrix at RT; with the test temperature increasing (less than fabrication temperature), the composite would possess a better bearing capacity because of the reduction of the thermal tensile residual stress, implying that the composite would have a gradual increase in strength with test temperature increasing. This analysis theoretically supports that the decreased thermally-induced residual stress in matrix is mainly responsible for the increased strength at temperatures ranging from 800 to 1200 °C in the current test environment.

### 3.4. Influence of ZrC Nanoparticles on Morphologies of Fractured Composites

In order to reveal the fracture mechanisms of composites during the flexural test at high temperature, it is essential to understand the stress distribution and the stress state during the flexural test. As we know, when an external load applied to the composite samples, the samples experienced different stress state in their bulk. The compression stress was applied to the upper part, while tension stress was applied to the lower part of the samples. Around the neutral axis of samples, the samples were subjected to a shear stress. Different stress state would result in the different failure modes.

In general, two failure modes, i.e., tensile failure and shear failure, occur during the three-point flexural test. If the fiber-matrix bonding is weak, the cracks induced by shear stress are preferred and start from the midsection of the composites. Then, the cracks are deflected and propagated along the fiber-matrix interfaces, so the stresses can be borne by the surviving fibers. When the fiber-matrix bonding is strong, the shear failure of the fibers would occur. The inter-laminar shear strengths were measured by DNS test, and they were 18.5 MPa and 11.8 MPa for CSZ0 and CSZ10, respectively. In a laminated composite, during the DNS test, the maximum stress, which is parallel to the fractured shear plane, should be proportional to the interfacial bonding stress, indicating that the inter-laminar shear strength should be closely related to the fiber-matrix bonding strength. From this, it can judge that the introduction of ZrC nanoparticles could decrease the fiber-matrix bonding strength.

Furthermore, the thermally-induced residual stresses should be taken into account for the analysis of mechanical properties. Due to the larger CTEs of SiC matrix and carbon fiber in the radial direction than that of carbon fiber in axial direction, the SiC matrix and carbon fiber in warp fiber bundle region would shrink more than that of weft fiber bundle region when the composites were cooled down from fabrication temperature to RT. Thus, the tension and compression residual stress would be accumulated in the warp and weft fiber bundle regions at temperature below 1410 °C [9,28], respectively. Such residual stresses would reach the largest magnitude at RT and their interaction would result in the accumulation of shear stress at the interface between warp and weft fiber bundle regions. Meanwhile, the tensile residual stress in warp fiber bundle region could be partially released by the generation of micro cracks as shown in Figure 4. During the flexural test, the composite would apply a tensile stress in its lower part. Thus, the applied tensile stress would superpose with the residual tensile stress in warp fiber bundle region. The superposed tension stress would accelerate the matrix cracking and finally result in the failure of composites. Generally, the micro cracks were firstly initiated from the matrix defects. When the tensile stress is perpendicular to the crack faces, the stress intensity in the cracks’ tip is very high, which induced the cracks to propagate perpendicularly to the weft fiber bundles’ region. If the fiber–matrix bonding is strong, the cracks can’t effectively deflect along the fiber-matrix interface. However, for the weak fiber-matrix bonding interfaces, with the assistance of shear stress, the cracks could easily deflect along the fiber-matrix interfaces [21].

**Figure 7:**
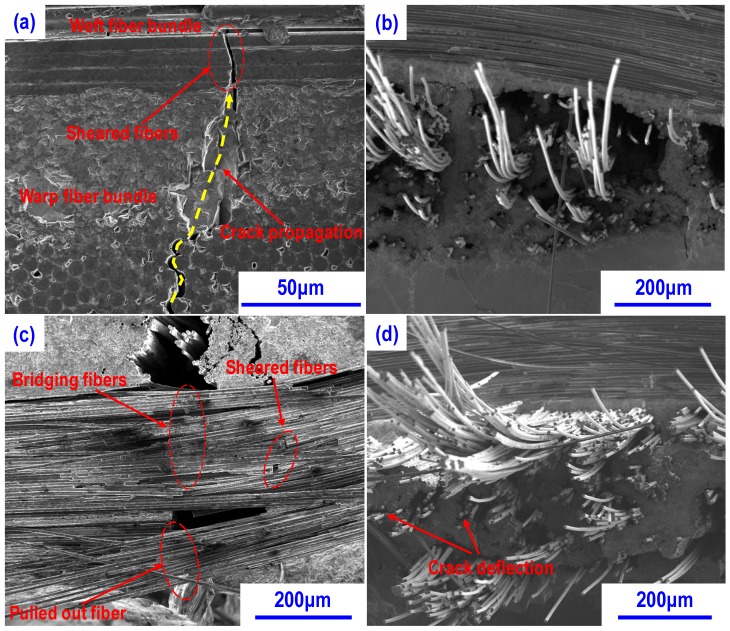
Morphologies of composites after flexural test at RT: (**a**) the side view of failed CSZ0 showing the sheared fibers by crack after passing through the matrix; (**b**) fracture surface of CSZ0; (**c**) the side view of failed CSZ10 showing the occurrence of interface debonding induced by crack propagation; (**d**) fracture surface of CZS10.

Figure 7 shows the morphologies of composites after flexural test at RT. Generally, if the fiber–matrix interface bonding is strong, the crack nucleation and deflection will be difficult at the fiber-matrix interface. As it can be seen from the side view of CSZ0 in Figure 7a, crack sheared the weft fiber bundles after passing through the matrix due to the strong interface bonding. Figure 7b shows the fracture surface of CSZ0 at RT. It can be seen that most of fibers were sheared and a small amount of fibers were pulled out. Also, the length of pulled out fibers is short. In contrast, in CSZ10, only a small amount of fibers were sheared, and the obvious fiber pullout and fiber bridging resulted from crack deflection were observed in weft fiber bundle region when the cracks passed through the matrix in warp fiber bundle region as shown in Figure 7c. Based on the observation on the fracture surface presented in Figure 7d, more fibers in CSZ10 were pulled out than that in CSZ0, indicating that the interfacial bonding in CSZ0 is stronger than that in CSZ10, which has been evidenced by the DNS test. And also, it can be seen that limited fibers were sheared and the length of pulled out fibers is long (Figure 7d). This phenomenon indicated that the interface bonding was modest in ZrC-modified composites. Thus cracks can’t shear the most of fibers and they have to deflect along the interfaces. The observed phenomenon is in agreement with the deformation behavior presented in Figure 5. It is well known that the strong interface bonding is not favorable to the improvement of mechanical properties of the ceramic matrix composites. Due to the weak interface bonding in CSZ10, the flexural strength of CSZ10 at RT is about 47% higher than that of CSZ0 as mentioned earlier.

The variation tendency of the mechanical properties with the test temperature could be ascribed to the change of residual stresses, the interfacial bonding and the matrix microstructure of the composites. Particularly, at low temperature, the high residual stresses play an important role in the damage of composites, such as matrix cracking and interface debonding [29]. As mentioned early, the residual stresses were proportional to the ΔT. With increasing test temperature, the residual stresses became correspondingly small.

**Figure 8:**
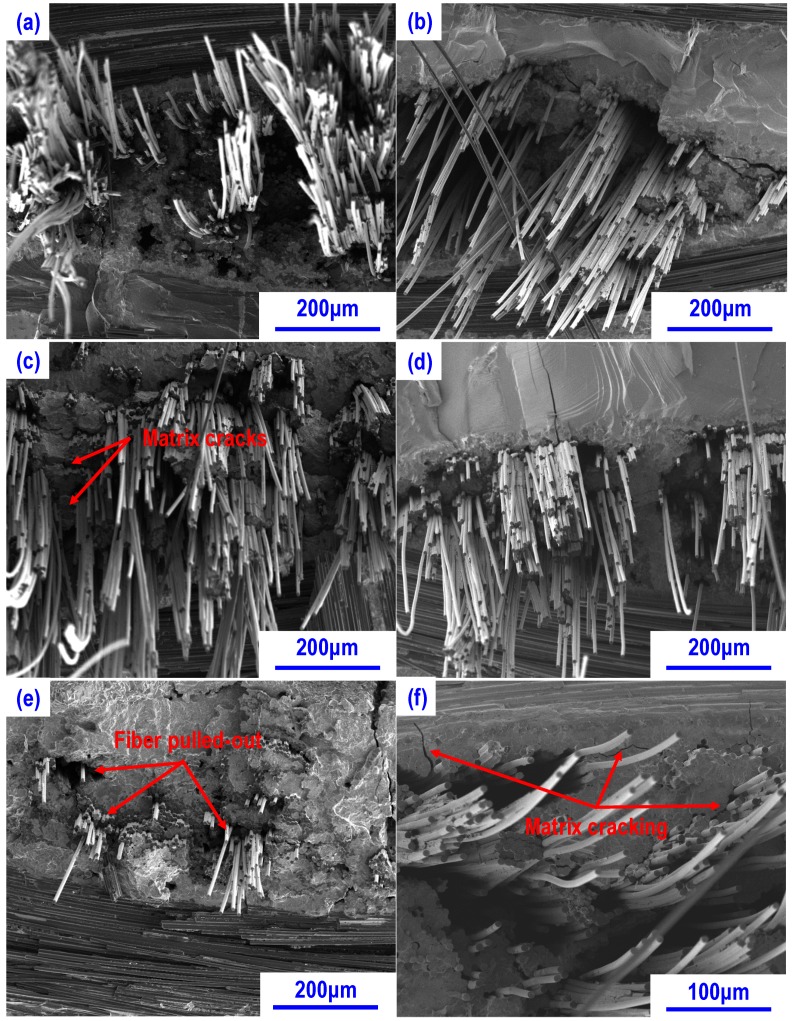
Morphologies of fracture surfaces at elevated temperatures: (**a**) CSZ0 at 800 °C; (**b**) CSZ10 at 800 °C; (**c**) CSZ0 at 1200 °C; (**d**) CSZ10 at 1200 °C; (**e**) CSZ0 at 1600 °C; (**f**) CSZ10 at 1600 °C.

Figure 8 shows the fracture surfaces of the composites after flexural test at high temperatures under vacuum. On the fracture surfaces of composites tested at 800 °C (Figure 8a,b), the amount and the length of pulled out fibers significantly increased for both CSZ0 and CSZ10 in comparison with those at RT (see Figure 7b,d), indicating that the interface bonding decreases to some extent. This was mainly attributed to the reduction of the residual stress in the composite. Meanwhile, from the observations in Figure 8a,b, the pulled-out fibers were mainly from the C/C region as shown on the surface morphologies (see Figure 4). This also indicated that the strong interface bonding is not helpful to transfer the load to fibers during the loading process. At 1200 °C, the fracture morphology of CSZ0 (Figure 8c) is obviously different from that of CSZ10 (Figure 8d). Especially, multiple matrix cracking and a step-like fracture surface were observed for CSZ0, indicating that the external load was effectively transferred from the matrix to fibers during the flexural test, which corresponded to a more pronounced pseudo-ductile fracture behavior. Moreover, the small amount of matrix was also stuck on the pulled out fibers. Such phenomenon might be related to the relatively high content of residual silicon. Probably, the pulled-out fibers could be attributed to the fracture of interface between SiC matrix and residual silicon. On the other hand, the softening of residual Si at 1200 °C should decrease the elastic modulus of composites as presented in Figure 6b. However, for CSZ10, few cracks could be observed in the matrix, indicating the network structure of SiC matrix could maintain the structure integrity of composites (see Figure 4c,d). It can be deduced from Figure 8c,d that CSZ10 has a better ability in keeping the structure integrity than CSZ0 at high temperature.

With the test temperature increasing to 1600 °C, the fracture morphologies of CSZ0 and CSZ10 were clearly different, as shown in Figure 8e,f. For CSZ0, compared with the composites tested at 1200 °C, the fracture surface is relatively even and very limited amount of pulled out fibers can be observed as shown in Figure 8e. There were cracks between fiber bundles and matrix, and the pulled-out length of fibers is short. In contrast, a large amount of pulled-out fibers with long length still can be observed as shown in Figure 8f. Furthermore, the matrix cracking could be clearly observed in both composites. The large amount of pulled out fibers would favor to the improvement of fracture behavior and mechanical properties as shown in Figure 5. The different fracture behavior and mechanical properties may be attributed to the different content of residual silicon and the distribution of SiC matrix. It was found that the amount of residual silicon in CSZ0 is higher than that of CSZ10, as shown in Figure 3 and Figure 4.

**Figure 9:**
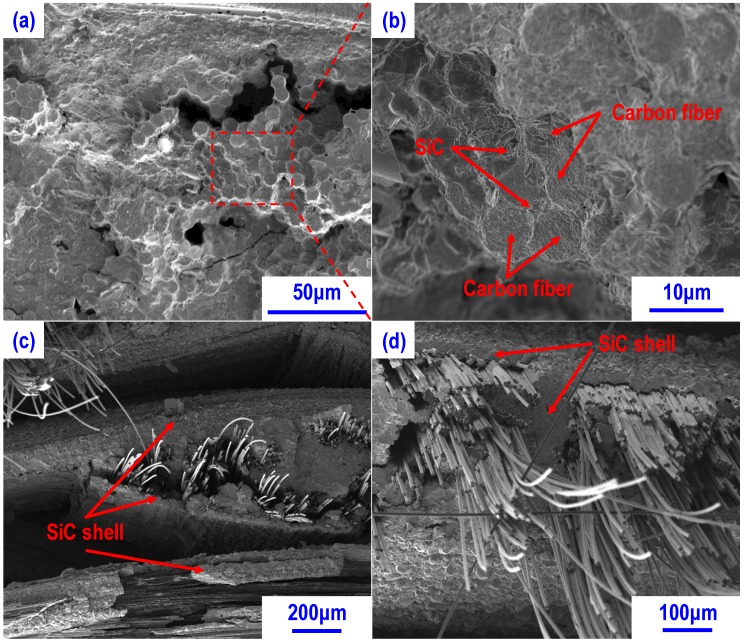
Microstructure of composites after flexural test under vacuum at 1600 °C: (**a**) CSZ0 showing the flat fracture surface almost no fiber pulled out; (**b**) high magnification image showing the closely bonded carbon fibers by the SiC formed during mechanical test; (**c**) microstructure of CSZ10 showing the SiC shell around carbon fibers; (**d**) high magnification image showing the pulled out carbon fibers wrapped with the SiC shell.

From Figure 9a,b (magnified view of composite tested at 1600 °C), it is easy to see that most of the carbon fibers were closely bonded by SiC. Such SiC matrix was mainly formed by the residual silicon melt with carbon fibers as well as the pyrolytic carbon during mechanical test at 1600 °C. Such reaction would be harmful to the carbon fibers and result in a strong fiber-matrix bonding, which are detrimental to the structure integrity and mechanical properties of composites [33]. However, in composite CSZ10, due to the small amount of residual silicon and homogeneous distribution of SiC matrix, carbon fibers avoided the serious damage and the SiC matrix formed a shell around the carbon fibers preventing the carbon fibers from further erosion as shown in Figure 9c,d. This could be the main reason why the flexural strength of the composite CSZ0 presented lower mechanical properties than those of CSZ10. On the other hand, the residual stress may also have an influence on the mechanical properties of composites. However, due to the melting of residual silicon and the small temperature difference between the test and the fabrication temperatures, the influence of thermal residual stress can be ignored in comparison with negative effect of residual silicon at 1600 °C. What’s more, the evaporation of residual Si at 1600 °C may also be responsible for the decrease of the mechanical properties [34]. Again, it is evidenced that the introduction of ZrC nanoparticles can improve the high-temperature mechanical properties of C-SiC composites.

## 4. Conclusions

The high-temperature mechanical properties of the ZrC-modified C-SiC composites fabricated by a hybrid process were measured from RT to 1600 °C under vacuum. The fracture features of composites before and after mechanical tests were characterized to reveal the influence mechanisms of ZrC nanoparticles on the high-temperature mechanical properties of C-SiC ceramic matrix composites. The main conclusions drawn from this work are summarized as follows:

(1) The composite exhibits typical pseudo-plastic behaviors at elevated temperatures. The flexural strength and flexural modulus show an asynchronous variation trend when the test temperature increases. The highest flexural strength is obtained at 1200 °C, while the elastic modulus at 800 °C is the highest. This is because the elastic modulus is more sensitive than the flexural strength to the defects produced during the loading. Furthermore, at all test temperatures, the ZrC-modified composite exhibits better mechanical properties than that of composite without nanoparticles. This indicates that the high-temperature performance of C-SiC composites can be improved by the introduction of ZrC nanoparticles.
(2) The high temperature mechanical properties are related to the consisting phase, the matrix microstructure and the thermally-induced residual stresses. Particularly, the thermally-induced residual stresses affect the internal stress state and the interface bonding of composite. The increase of strengths from RT to 1200 °C is mainly attributed to the decrease of the thermally-induced residual stresses. The decreased strength at 1600 °C is mainly associated with the residual silicon, which would cause the new defects and the degradation of carbon fibers.
(3) Microstructure characterization for the composite without ZrC nanoparticles reveals that the residual silicon would react with carbonaceous materials at 1600 °C to form a strongly bonded fiber-matrix interface, which is detrimental to the mechanical properties of the composite. Therefore, the better mechanical properties for the ZrC-modified composite are attributed to the small content of residual silicon and the formation of continuous SiC network structure in the matrix, which are beneficial to the structure integrity and mechanical properties of composite.
